# Supplementary material for: Causal Associations between Vitamin D Levels and Psoriasis, Atopic Dermatitis, and Vitiligo: A Bidirectional Two-Sample Mendelian Randomization Analysis
Source: Nutrients. 2022 Dec 11;14(24):5284. doi: 10.3390/nu14245284 (PMC9785064; doi:10.3390/nu14245284)
Supplement: Supplementary file 1 [file nutrients-14-05284-s001.zip › Supplementary_materials_figures_1207.pdf]

Causal Associations Between Vitamin D Levels and Psoriasis, Atopic Dermatitis, and Vitiligo: A Bidirectional Two-Sample Mendelian Randomization Analysis

Supplementary materials

Supplementary Tables

Table S1 GWAS data sources used in the present study.

Table S2 The characteristics of instrumental variables (IVs) (LD clumping r2<0.001).

Table S3 Mendelian randomization analysis results for IVs with LD $r^2 < 0.001$.

Supplementary Figures

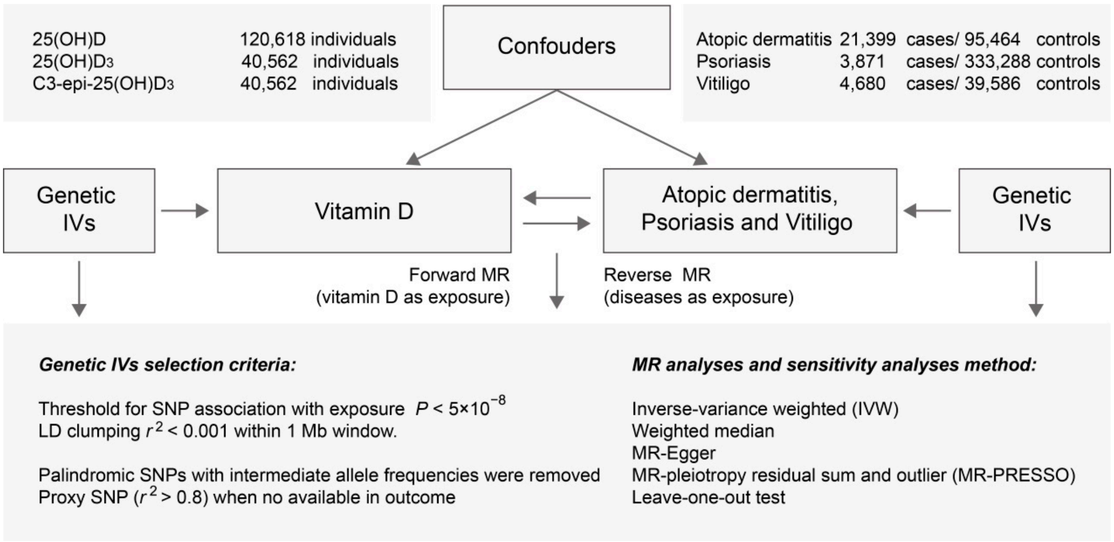

Figure S1 Study design

We performed a bidirectional MR analysis to estimate the causal associations between vitamin D levels and the risk of atopic dermatitis, psoriasis, and vitiligo. We identified genetic IVs from the GWAS for vitamin D levels, atopic dermatitis, psoriasis, and vitiligo, and performed MR analyses using four approaches. Abbreviations: 25(OH)D, 25-hydroxyvitamin D; 25(OH)D_3, 25-hydroxyvitamin D_3; C3-epi-25(OH)D_3, C3-epimer of 25-hydroxyvitamin D_3; MR, Mendelian randomization; IVs, instrumental variables; LD, linkage disequilibrium.

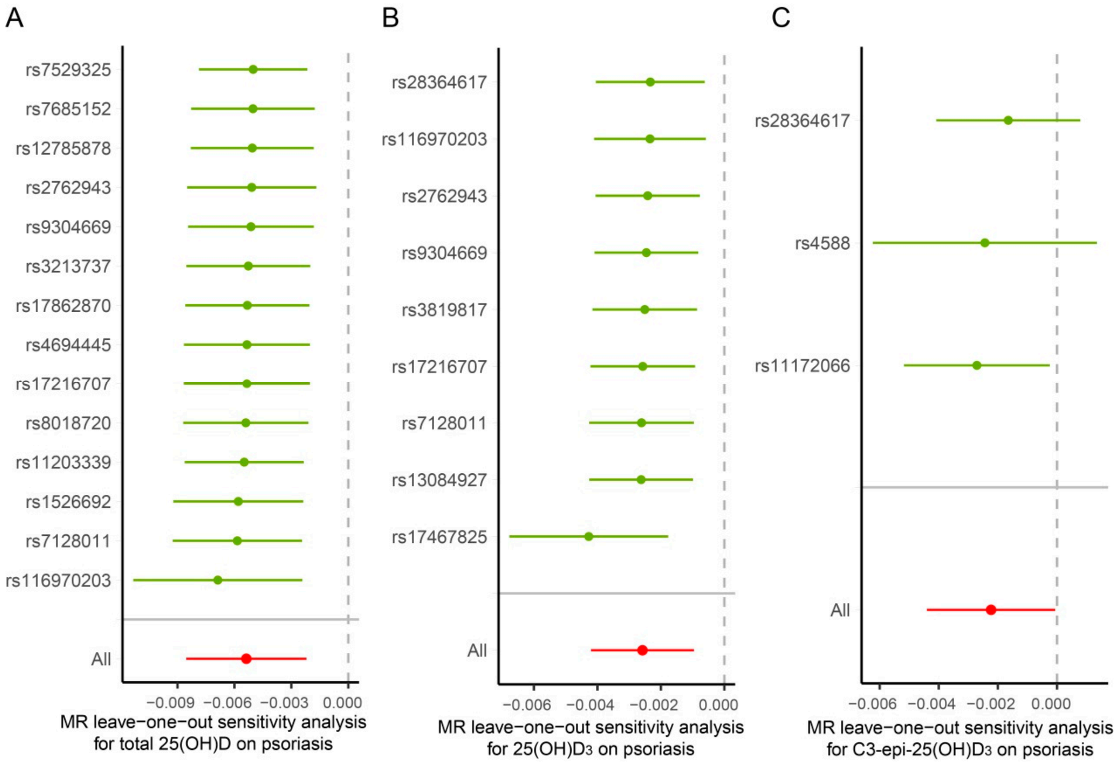

Figure S2 Leave-one-out sensitivity analysis for MR analysis.

The leave-one-out plots for the causal effects of (A) 25(OH)D, (B) 25(OH)D_3, (C) C3-epi-25(OH)D_3 on the risk of psoriasis. Each row represents the causal effect of the exposure on the outcome estimated in the inverse-variance weighted method after excluding the corresponding genetic variant on the y-axis. Abbreviations: 25(OH)D, 25-hydroxyvitamin D; 25(OH)D_3, 25-hydroxyvitamin D_3; C3-epi-25(OH)D_3, C3-epimer of 25-hydroxyvitamin D_3; MR, Mendelian randomization.

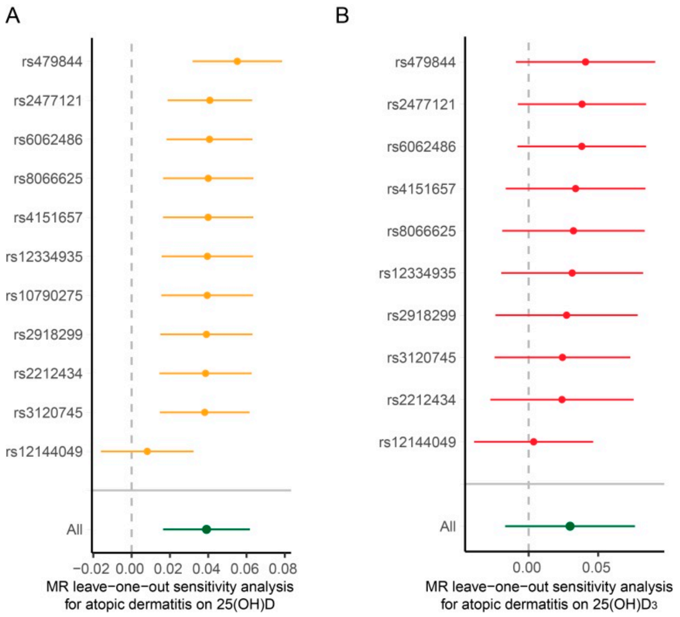

Figure S3 Leave-one-out sensitivity analysis for reverse MR analysis.

The leave-one-out plot for the causal effects of atopic dermatitis on (A) 25(OH)D and (B) 25(OH)D_3. Each row represents the causal effect of the exposure on the outcome estimated in the inverse-variance weighted method after excluding the corresponding genetic variant on the y-axis. Abbreviations: 25(OH)D, 25-hydroxyvitamin D; 25(OH)D_3, 25-hydroxyvitamin D_3.
